# Vaccinia Virus Infections in Martial Arts Gym, Maryland, USA, 2008

**DOI:** 10.3201/eid1704.101010

**Published:** 2011-04

**Authors:** Christine M. Hughes, David Blythe, Yu Li, Ramani Reddy, Carol Jordan, Cindy Edwards, Celia Adams, Holly Conners, Catherine Rasa, Sue Wilby, Jamaal Russell, Kelly S. Russo, Patricia Somsel, Danny L. Wiedbrauk, Cindy Dougherty, Christopher Allen, Mike Frace, Ginny Emerson, Victoria A. Olson, Scott K. Smith, Zachary Braden, Jason Abel, Whitni Davidson, Mary Reynolds, Inger K. Damon

**Affiliations:** Author affiliations: Centers for Disease Control and Prevention, Atlanta, Georgia, USA (C.M. Hughes, Y. Li, C. Dougherty, C. Allen, M. Frace, G. Emerson, V.A. Olson, S.K. Smith, Z. Braden, J. Abel, W. Davidson, M. Reynolds, I.K. Damon) ;; Maryland Department of Health and Mental Hygiene, Baltimore, Maryland, USA (D. Blythe);; Maximed Associates, Silver Spring, Maryland, USA (R. Reddy);; Montgomery County Department of Health and Human Services, Silver Spring (C. Jordan, C. Edwards, C. Adams, H. Conners, C. Rasa, S. Wilby, J. Russell, K.S. Russo);; Michigan Department of Community Health, Lansing, Michigan, USA (P. Somsel);; Warde Medical Laboratory, Ann Arbor, Michigan, USA (D. Wiedbrauk)

**Keywords:** Vaccinia, smallpox vaccine, martial arts, sports, viruses, Maryland, dispatch

## Abstract

Vaccinia virus is an orthopoxvirus used in the live vaccine against smallpox. Vaccinia virus infections can be transmissible and can cause severe complications in those with weakened immune systems. We report on a cluster of 4 cases of vaccinia virus infection in Maryland, USA, likely acquired at a martial arts gym.

Vaccinia virus (VACV) is the virus used in the live vaccine against smallpox. Smallpox was declared eradicated by the World Health Organization in 1980 (*1*), and routine childhood smallpox vaccination ceased after 1972 in the United States. Since 2002, smallpox vaccinations have again been administered to some military personnel and health care workers, and they continue to be recommended for laboratory workers who work with nonattenuated orthopoxviruses (*2*). VACV infections are transmissible and can cause severe complications in those with weakened immune systems (*3*). We report a cluster of community-acquired VACV infections at a martial arts gym in Maryland, USA.

## Case-Patients

**Figure 1:**
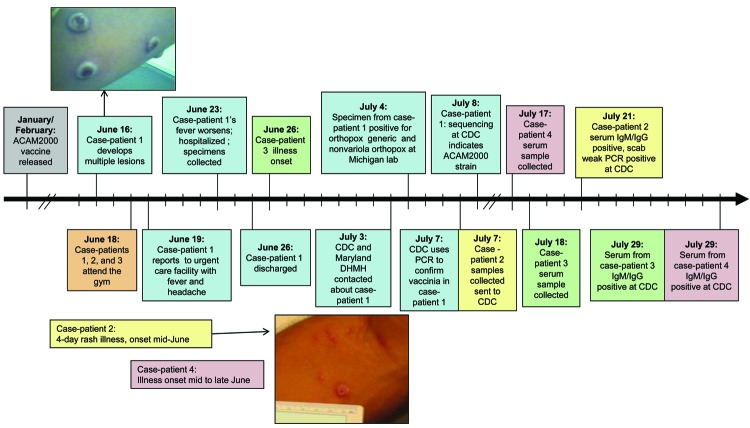
Timeline of the vaccinia cluster, Maryland, USA, 2008. The photo of case-patient 1’s skin lesions was taken on ≈day 8 of illness (courtesy of R. Reddy). The photo of case-patient 2’s skin lesions was taken ≈3 weeks after lesion onset (courtesy of K. Russo). Blue shading, case-patient 1; yellow shading, case-patieint 2; green shading, case-patient 3. CDC, Centers for Disease Control and Prevention; Ig, immunoglobulin; DHMH, Department of Health and Mental Hygiene.

In July 2008, the Michigan Department of Community Health (MDCH) Bureau of Laboratories reported a suspected orthopoxvirus infection to the Centers for Disease Control and Prevention (CDC). In the affected person, a 26-year-old male resident of Maryland , multiple pustules had developed on the arm, chin, and back of the knee on June 16 (Table; case-patient 1). He sought treatment after a fever and headache developed on June 19 and was advised to go to an emergency room if his fever worsened (Figure 1). His condition did not improve, and he was hospitalized in Maryland on June 23. His lesions were umbilicated pustules ≈0.5 cm in diameter with similar morphologic features (Figure 1). The cause of his illness remained undetermined, and he was discharged on June 26 after defervescence. Contact precautions were employed during the hospitalization; however, the patient was not isolated in a negative-pressure isolation room.

Lesion samples were collected on June 24 and forwarded by the Maryland hospital to a virology reference laboratory in Michigan for testing. The samples were negative by PCR for varicella, adenovirus, and herpes simplex virus. Cytopathic effect suggestive of an orthopoxvirus was noted in MRC-5, A549, and primary rhesus monkey kidney cells, and the samples were forwarded to the MDCH laboratory for further testing. On July 4, the MDCH laboratory confirmed the presence of orthopoxvirus DNA in the lesion sample using an orthopoxvirus (nonvariola) and an orthopoxvirus generic real-time PCR (*4*). The specimens were sent to CDC for confirmation and virus species identification. Real-time PCRs designed to differentiate orthopoxvirus species were performed, and samples were positive for VACV DNA but not for monkeypox virus DNA (*5*) (Table).

**Table:** Characteristics of 4 cases of vaccinia infection at a martial arts gym, Maryland, USA, 2008*

Case-patient no.†	Age, y/sex	Date of onset	Rash features	Initial diagnosis	PCR	Serologic results
1	26/M	Jun 16	Pustules on face, arm, back of knee	Unknown viral exanthem	+	NA
2	28/M	Mid–late Jun	Vesicles on right forearm	None	Weak +	IgM+ (0.243), IgG+ (0.116)
3	31/M	Jun 25	Unknown presentation	MRSA‡	NA	IgM+ (0.389), IgG+ (0.227)
4	31/M	Late Jun/early Jul	Unknown presentation	MRSA‡	NA	IgM+ (0.137), IgG+ (0.2195)

*Ig, immunoglobulin; NA, not applicable; MRSA, methicillin-resistant *Staphylococcus aureus*. †For purposes of this investigation, a case-patient is defined as a person whose clinical samples are positive for vaccinia virus DNA by PCR, or a person with lesions or a rash (macular, papular, vesicular, or pustular) and whose serum sample was positive for anti-orthopoxvirus IgM antibodies (indicative of a recent orthopoxvirus exposure). No case-patients had received a previous smallpox vaccination. ‡Clinically diagnosed, no laboratory confirmation.

The Maryland Department of Health and Mental Hygiene was contacted on July 3 and, in collaboration with the Montgomery County Department of Health and Human Services, began an investigation. The patient was asked whether he recently received smallpox vaccination or had history suggestive of exposure to orthopoxviruses such as monkeypox virus (i.e., contact with animals, recent international travel). He reported having neither; however, his wife and child had returned from a trip to Brazil 2 weeks before his illness. Human VACV infections caused by contact with infected dairy cattle occur in regions of Brazil (*6*). His wife only visited an urban area in Brazil, reported no contact with farm animals, and reported no fever or rash.

**Figure 2:**
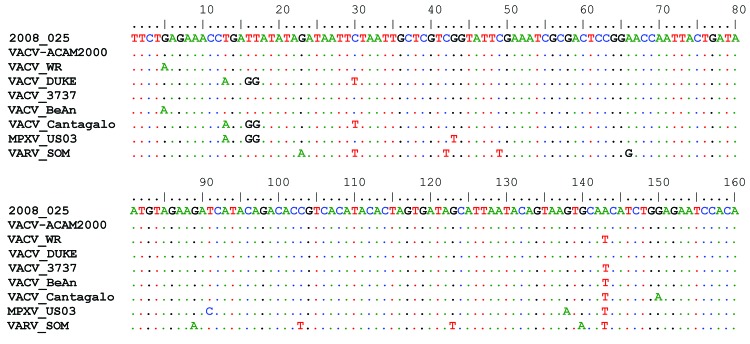
Partial DNA sequence alignment of the hemagglutinin gene. Case-patient 1’s isolate sequence is displayed at the top (2008–025). Dots in the alignment indicate identical nucleotides at that position. The reference sequences shown: current smallpox vaccine strain (VACV_ACAM2000), a commonly used laboratory vaccinia strain (VACV_WR), Dryvax vaccinia strains (VACV_Duke and VACV_3737), natural Brazilian vaccinia isolates (VACV_BeAn and VACV_Cantagalo), a 2003 US monkeypox outbreak isolate (MPXV_US03), and a variola virus isolate (VARV_SOM). Reference GenBank accession nos., AY313847, NC_006998, DQ439815, DQ377945, DQ206442, AF229247, DQ011157, and DQ437590, respectively.

Sequence analysis of a 160-bp fragment of the hemagglutinin gene from the virus isolate was performed at CDC to determine whether the VACV strain originated from smallpox vaccine or from a strain that occurs naturally in Brazil. The isolate matched the strain used in the ACAM2000 smallpox vaccine and was distinctive from known Brazilian VACV (Figure 2). ACAM2000 is the second-generation smallpox vaccine, which replaced Dryvax in January/February 2008 (*7*).

The patient reported belonging to a martial arts gym; he reported having several military personnel as recent sparring partners before the onset of his illness. He also reported that a recent sparring partner had exhibited a rash around the same time. This person, a 28-year-old man (case-patient 2), was contacted and described having a 4-day rash on his right forearm in mid to late June with no systemic symptoms (Figure 1). Samples of his serum, collected 2–3 weeks after lesion onset, were sent to CDC for testing and showed modestly elevated levels of anti-orthopoxvirus immunoglobulin (Ig) M antibodies (Table). Scab material tested at CDC was weakly positive for orthopoxvirus DNA by using the orthopoxvirus nonvariola and orthopoxvirus (generic) PCRs.

In the absence of an explanation for these 2 VACV (ACAM2000) infections, Maryland public health officials launched an investigation at the gym to identify additional cases and pinpoint the source of infection. Approximately 400 surveys were distributed to gym members through email and by hand at the gym. Members were asked whether they had any recent skin lesions similar to those shown in an attached photo. They were asked whether they had recently received a smallpox vaccination or had contact with someone recently vaccinated.

Ninety-five gym members responded to the survey. Several reported having received a smallpox vaccination previously, but none reported vaccination within the prior 2 months. Thirteen gym members reported skin lesions or rash but no recent smallpox vaccination. Two of these persons (case-patients 3 and 4) were clinically diagnosed with methicillin-resistant *Staphylococcus aureus* (MRSA) by health care providers in late June and early July. Both attended the gym on the same day as case-patient 1 (Figure 1). Serum samples were collected from these men 3–4 weeks after lesion onset and sent to CDC for testing. Both had elevated levels of anti-orthopoxvirus IgG and IgM antibodies, indicative of a recent exposure (Table).

Maryland public health officials reviewed cleaning protocols at the gym. They determined that equipment and pads were cleaned at least twice daily (stemming from a concern about MRSA transmission) and that appropriate cleaning products were being used.

CDC identified 5 civilian clinics that had received ACAM2000 vaccine since late February in the Maryland area. These clinics reported having vaccinated 65 persons; none were members of the martial arts gym. The Military Vaccine Agency (Milvax) cross-checked its list of recent military vaccinees against the gym member list since late February. Although several of those identified as being vaccinated had an association with the gym, they were either not currently gym members or were not at the gym during this period. The source of virus introduction into the martial arts gym remains unknown. No further infections have been identified among gym members or health care workers exposed to case-patients.

## Conclusions

This cluster of community-acquired VACV infection was possibly the result of sequential person-to-person spread of virus through direct physical contact, although transmission through fomites cannot be ruled out. The ultimate source-person responsible for introducing the virus into the gym was not identified, but given the limited time that ACAM2000 had been available to providers in the region (late February 2008), the most likely source was a recent vaccinee. None of the current gym members were known to have been vaccinated within the 4 weeks before illness onset of the first case-patient. Unrecognized transmission of VACV among gym members may have been ongoing over several months.

Multiple cases of VACV infection caused by secondary transmission have been noted recently (*8**–**13*). Materials such as towels and bedding used by the vaccinee should be treated as potential fomites and should not be shared with others (*14*). To our knowledge, this is the first reported cluster of community acquired VACV in which an obvious source-person was not identified. This cluster highlights the need to reinforce transmission precautions to recent vaccinees and indicates that physicians should include VACV infections on the differential of vesiculopustular rash lesions and take appropriate infection control precautions, even in the absence of a known exposure to smallpox vaccine.
